# Artificial intelligence supported patient self-care in chronic heart failure: a paradigm shift from reactive to predictive, preventive and personalised care

**DOI:** 10.1007/s13167-019-00188-9

**Published:** 2019-11-22

**Authors:** Matthew Barrett, Josiane Boyne, Julia Brandts, Hans-Peter Brunner-La Rocca, Lieven De Maesschalck, Kurt De Wit, Lana Dixon, Casper Eurlings, Donna Fitzsimons, Olga Golubnitschaja, Arjan Hageman, Frank Heemskerk, André Hintzen, Thomas M. Helms, Loreena Hill, Thom Hoedemakers, Nikolaus Marx, Kenneth McDonald, Marc Mertens, Dirk Müller-Wieland, Alexander Palant, Jens Piesk, Andrew Pomazanskyi, Jan Ramaekers, Peter Ruff, Katharina Schütt, Yash Shekhawat, Chantal F. Ski, David R. Thompson, Andrew Tsirkin, Kay van der Mierden, Chris Watson, Bettina Zippel-Schultz

**Affiliations:** 1 University College of Dublin, Catherine McAuley Education & Research Centre, Mater Misericordiae University Hospital, Nelson Street, Dublin, 7 Ireland; 2 Department of Cardiology, Maastricht University Medical Center, PO Box 5800, 6202AZ Maastricht, The Netherlands; 3 Department of Cardiology, University Hospital Aachen, Pauwelsstrasse 30, 52074 Aachen, Germany; 4 Thomas More University of Applied Science, Kleinhoefstraat 4, 2240 Geel, Belgium; 5 Belfast Health and Social Care Trust, A Floor, Belfast City Hospital, Lisburn Rd, Belfast, BT9 7AB UK; 6 Queen’s University Belfast, 97 Lisburn Rd, Belfast, BY9 7BL UK; 7 Radiological Clinic, Universitätsklinikum Bonn, Excellence University of Bonn, Sigmund-Freud-Str. 25, 53127 Bonn, Germany; 8 Sananet Care BV, Rijksweg Zuid 37, 6131AL Sittard, Netherlands; 9 RIMS bvba, Bollestraat 75, 3090 Overijse, Belgium; 10 German Foundation for the Chronically Ill, Alexanderstrasse 26, 90762 Fürth, Germany; 11 Nurogames GmbH, Schaafenstrasse 25, 50676 Cologne, Germany; 12 Exploris AG, Tödistrasse 52, 8002 Zürich, Switzerland

**Keywords:** Heart failure, Artificial Intelligence, Predictive preventive personalised participatory medicine, Individualised patient profile, Patient engagement, Information and communications technology, Healthcare economy, Patient stratification, Diabetes, Comorbidities, Healthcare digitalisation, Therapy monitoring, Professional interactome, Multi-level diagnostics, Disease modelling, Integrated care, Medical ethics, Societal impact

## Abstract

Heart failure (HF) is one of the most complex chronic disorders with high prevalence, mainly due to the ageing population and better treatment of underlying diseases. Prevalence will continue to rise and is estimated to reach 3% of the population in Western countries by 2025. It is the most important cause of hospitalisation in subjects aged 65 years or more, resulting in high costs and major social impact. The current “one-size-fits-all” approach in the treatment of HF does not result in best outcome for all patients. These facts are an imminent threat to good quality management of patients with HF. An unorthodox approach from a new vision on care is required. We propose a novel predictive, preventive and personalised medicine approach where patients are truly leading their management, supported by an easily accessible online application that takes advantage of artificial intelligence. This strategy paper describes the needs in HF care, the needed paradigm shift and the elements that are required to achieve this shift. Through the inspiring collaboration of clinical and high-tech partners from North-West Europe combining state of the art HF care, artificial intelligence, serious gaming and patient coaching, a virtual doctor is being created. The results are expected to advance and personalise self-care, where standard care tasks are performed by the patients themselves, in principle without involvement of healthcare professionals, the latter being able to focus on complex conditions. This new vision on care will significantly reduce costs per patient while improving outcomes to enable long-term sustainability of top-level HF care.

## Introduction

Heart failure (HF) is one of the most prevalent, complex and costly forms of cardiovascular disease [1]. In North-West Europe HF affects 3.6 million people and is predicted to increase to more than 5 million in 2025 [2]. Estimated costs vary significantly between different studies but may be as high as more than €10,000 per patient each year and life-time costs from HF diagnosis to death of > €100,000 [3, 4]. Without active participation of patients in their care processes, the burden of HF on healthcare labour and costs is unsustainable.

Digital medicine offers an important contribution to solve this socially urgent problem; however, existing eHealth solutions are not integrated in the care processes and value chains. Currently, HF eHealth products are stand-alone add-ons to standard treatment rather than supporting patients effectively with their self-care. Next-generation solutions will take advantage of artificial intelligence (AI) and gaming to provide interactive educational and decision-making support mechanisms enabling patients to engage in self-care. These solutions are expected to reduce costs, improve quality of care and ensure access to healthcare regardless of time, location and staff scarcity.

This strategic paper put forth by the Patient Self-care uSing eHealth In chrONic Heart Failure (PASSION-HF) consortium presents the evidence to support this paradigm shift in HF care. Using digital therapeutics powered by AI (e.g. decision support engine, interactive physician avatar interface, serious gaming tools, self-learning feedback system, patient coaching), the objective of PASSION-HF is to develop a virtual ‘doctor at home’ system called *Abby*. Abby will be the enabler to *predictive, preventive and personalised medicine* (PPPM) or *effective HF patient self-care* according to HF-guidelines, in consideration of comorbidities and ensuring safe prescribing and management of medication.

## Necessity for a paradigm shift in the care of HF

### HF prevalence

Driven by an ageing population, HF remains a growing pandemic estimated at almost 2% of the adult population [5, 6]. The prevalence of HF generally doubles for each decade of life from < 1% for those aged under 40 to > 10% for those aged over 75 [7]. Due to advances in medical treatment and device therapy, HF incidence rates are decreasing whilst prevalence rates are increasing [6, 8].

### HF hospitalisations and mortality

Due to worsening symptoms and the advanced age of HF patients suffering severe comorbidities, HF is also associated with high rates of hospital admissions and outpatient visits. Between 1979 and 2004, the rates of HF hospitalisation in the USA increased from 219 to nearly 400 per 100,000 HF patients [9]. The number of hospitalisations for HF in other European countries like the Netherlands, Scotland and Sweden reached its maximum in the 1990s [10]. In the UK, 5% of admissions from the emergency department to the hospital have been linked to HF. Moreover, HF hospitalisation rates have been predicted to increase by more than 50% over the next two decades [7]. HF is one of the five most common causes of death worldwide [11]. Significant improvements in current healthcare must occur otherwise the burden of HF will continue to rise exponentially [6]. If current trends continue, it has been estimated that by the year 2030 more than 8 million deaths will be attributed to HF [11].

### Quality of life and satisfaction

The impact of HF is evident not only in rising numbers but also in declining patient-reported quality of life [11]. Severe symptoms, like dyspnoea or fatigue, and increasing accompanying comorbidities have serious implications for health-related quality of life (HRQoL). HRQoL refers to the impact of an illness and its treatment on the ability to live a fulfilling life from the patient’s perspective [12]. Self-rated health is an important part of HRQoL that reflects physical and mental wellbeing and is directly associated with the progression of HF [12]. Patient satisfaction is another important aspect, which is increasingly considered to be an indicator of HF quality of care [13]. Of significance to those with HF, patient satisfaction can be divided in three sections: overall level of satisfaction with healthcare services, satisfaction with the treatment and satisfaction with medication [14]. Patient satisfaction has also been linked to cost-effectiveness. Studies have found that HF patients who are more satisfied with their treatment had fewer doctor and specialist appointments, reported fewer complaints and had better adherence to therapy [14].

An important, still underrated aspect in this regard is the role of family carers, who feel a responsibility to be available almost around the clock and therefore give up social activities and even work [15]. Emotional bonds make it difficult for carers to take a step back and consider their personal health and quality of life. In this way, HF not only affects patients but also their carers who report severe physical and psychological effects [16].

### HF costs to society

HF costs are immense, meaning affordability is a problem across various healthcare systems and countries in Europe and around the world [17, 18]. The main driver being an increase in chronic disease and multimorbidity due to an ageing population [19]. As a consequence, healthcare systems face a new challenge; treating multimorbid patients, whilst attempting to keep costs down [20, 21]. HF is not only the fastest growing cardiovascular condition worldwide [7] but also has the highest hospitalisation rates; hence, costs for HF are among the highest for all chronic conditions [22]. Estimates suggest that HF results in 57 billion Euro in direct care costs and 38 billion Euro in indirect costs globally [23]—the equivalent to approximately 2% of total healthcare costs in Europe and North America, over 60% of which are for inpatient care [4]. Of significance, there have been alarming projections of total HF costs increasing by 127% between 2012 and 2030 [1]. The UK’s Centre for Economics and Business Research concluded that the cost of cardiovascular disease in Europe will rise from €100 billion to approximately €120 billion over the next 10 years [7]. Indirect costs of HF also need to be considered, though less explored, as they contribute extensively to the overall burden caused by HF [24]. For example, in Poland, a substantial public finance burden of HF was calculated at €871.9 million in 2012, which increased to €945.3 million in 2015 [24].

Socioeconomic status strongly influences risk for HF and overall mortality [11, 25]. HF disproportionally affects lower-income, poorly educated patients who have less access to healthcare. More educated patients with higher-income usually report lower morbidity from HF and other chronic diseases such as diabetes or hypertension [26]. Of importance, socioeconomic deprivation has been shown to be a powerful independent predictor of HF development and adverse outcomes [11], with some HF studies associating lower socioeconomic status with poorer survival [27, 28]. Last but not least, the rapidly increasing problem of staff shortages, both for qualified nurses and doctors, in North-West Europe (NWE) is an additional cause for serious concern. Ultimately, the present state of HF healthcare is not sustainable.

### Current state of HF care—a HF case report

Mr. Johnson (78 years old), a patient with HF, lives with his wife in the countryside. He was an independent farmer, but since his heart attack 3 years ago, his physical functioning has declined. His heart function is impaired, he suffers from atrial fibrillation, decreased kidney function and has anaemia. Cognitively he is good. The children are not able to help because they live at great distance. The couple is affiliated with a GP practice several villages away, which is not easy to get there as they do not drive a car anymore and public transport is poor. The hospital is far away in the city, and for clinical visits, they must travel by taxi that is hardly affordable.

Mr. Johnson has many doubts about his disease. It is not clear whether he gets the adequate medication and dosages. His GP is overloaded and has difficulties helping him sufficiently because of the complexity of the disease and his comorbidities. He was seen once quickly by the cardiologist at the outpatient clinic for the required examinations. Mr. Johnson was left with unanswered questions and inadequate treatment. His exercise tolerance remained very limited and sometimes he could not sleep because of breathlessness. His GP was not able to improve his condition while waiting for more than three months before a further appointment with the cardiologist. His condition deteriorated significantly, and once he developed breathlessness at rest he was admitted to the hospital. Although some additional medication helped, he still had limited mobility and many questions arose. An appointment with the HF nurse is due in 6 months; however, the distance and the difficulty to consult closely with his care providers make him and his wife anxious. What should he do if the physical limitations increase again and he starts to retain fluid? Is there a timely intervention? Who accompanies him sufficiently in this?

**Fig. 1:**
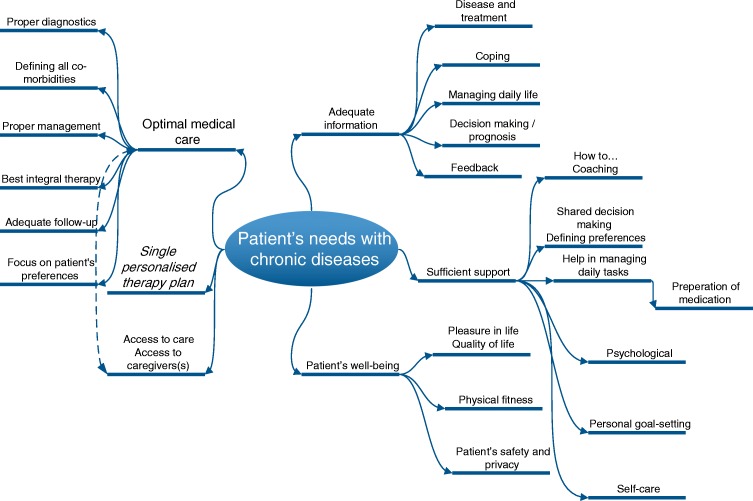
Important elements of patient needs in chronic diseases such as HF. They include different elements of adequate information, optimal medical care, single personalised treatment plan, access to care, adequate information, sufficient support and sufficiently considering patient well-being to enable self-care supported by eHealth including artificial intelligence

This case report illustrates the fears and needs of HF patients and commonly the lack of healthcare services and support. Access to high quality care is restricted in many countries or regions. Actions taken to prevent excess costs are not only insufficient, they act as disablers to care that is personalised, predictive and preventive. In fairness, the needs of HF patients are often significantly broader than outlined in this paragraph and depicted in Fig. 1. Unless extensive and immediate advancements in healthcare take place, it is unlikely that the needs of the HF patient will be met.

When healthcare remains in the status quo future healthcare of patients with chronic diseases in general, and HF in particular, will be scarce [29]. Initiatives so far have not brought sufficient solutions [30]. Current organisation of HF care is not optimal and the capacity of health professionals is increasingly limited, resulting in a lack of focused effort on the most urgent cases. Furthermore, complexity arises as most HF patients suffer from a range of comorbidities. Any combination of these complicates diagnosis, therapeutic decision-making and can require identifying a different care regimen (e.g. drug compliance, adverse reactions). Change is necessary; a more personalised approach to HF care is warranted.

## A new culture in healthcare—the paradigm shift

Healthcare systems around the globe continue to accumulate a number of persisting and even sharpening problems. A pessimistic scenario considers epidemics of chronic non-communicable disease with severe economic consequences to society. Additionally, although personalised treatment approaches receive more appreciation by patients and recognition by medical doctors, they lead to significantly increased costs to healthcare as a whole. Consequently, there are, on the one hand, rapidly growing subpopulations of chronically diseased people and, on the other hand, an ever-increasing demand for more personalised medical services.

### Key characteristics of the new predictive, preventive and personalised approach

Key characteristics of the *new culture to be developed in healthcare* have been thoroughly analysed and are well presented in the book “Modern Hospital” released by Springer in 2019 [31]. The central concepts can be summarised as follows:

- Predictive health versus currently applied reactive ‘disease care’ approach
- Current unmet needs of patient cohorts to be considered in a long-term manner via application of advanced technologies
- Optimised healthcare economy via cost-effective medical services
- New dimension of professional interests and tasks
- New scale of the knowledge-integration
- Highly motivated technological innovation
- Highly motivated cross-professional cooperation
- Active participation of patients in the healthcare processes.

The paradigm shift from a delayed reactive type of medicine to PPPM considers the conceptualised difference between ‘disease care’ (cost-ineffective reactive treatments of chronic pathologies that contain a large portion of preventable events such as a costly hospitalisation in acute cases) and ‘healthcare’ (i.e. targeted preventive measures aimed at maintaining health capacity of individuals, even in the presence of chronic diseases such as HF) approaches, including impacts on ethical and economical aspects of medical services [46]. What is recommended to reach the goal of healthcare?

*Individualised patient profiling* is essential for patient stratification, characterisation of individual predisposition and creation of treatments tailored to the person [32]. Profiling comprises the patient history in full detail including information about comorbidities, and aspects such as behavioural factors (e.g. nutrition, patient preferences) and individual skills (e.g. technology related).

*Multi-level diagnostics* is a robust platform for PPPM strategies which includes patient-specific questionnaires, medical imaging, molecular biological characterisation (e.g. comprehensive biomarker panels) and novel eHealth based diagnostic tools (e.g. novel multilevel sensors) as well as the combination and integration of all of them.

*Professional interactomes amongst relevant stakeholder groups including patients* should be designed in order to create a multi-professional approach with multidisciplinary expertise in the personalised treatment of HF and accompanying comorbidities. Importantly, patients must be a central part of these interactomes in order to create the required new culture in healthcare.

*New educational programmes* are required to improve the knowledge of professionals, patients and the general population of the new PPPM approach. Programmes should contain detailed information about the above listed points explaining: why the paradigm shift is needed; what are the enabling instruments; why multi-level diagnostics is more advanced compared to currently available approaches; how the healthcare interactome can be designed in the most optimal way; and what are the benefits for patients and healthcare professionals [33].

### The digital transformation in care

Digital transformation is profoundly and rapidly impacting our daily life, including social, economic and political aspects. Digital transformation has affected our daily communications and interactions. In medicine, digitalisation has altered disease: monitoring, education and personalised coaching, diagnostic and prediction tools, consultation services, therapeutic strategies, and screening and prevention. Therefore, in a time of demographic, social and medical change, the health professional perspective is essential to guide the implementation of new digital technology [34]. Healthcare professionals are those best placed to identify disease-related barriers and consider possible patient-centred solutions [35]. Led by healthcare professionals and powered by AI, daily routine medical information will be integrated into patient support systems, thus facilitating needs-led patient-doctor interactions and therapeutic decision-making of strategic relevance.

## The HF paradigm shift to PPPM

### Heterogeneity, risk factors and personalised profiles of patients with HF

HF is a complex disease entity, being typified by a common ‘downstream’ clinical syndrome but with a broad range of ‘upstream’ structural, functional and biochemical phenotypes as well as diverse disease courses and prognoses [36]. Assessment and treatment of patients presenting with stable or decompensated HF must be performed in a manner tailored to the patient’s individual phenotype [37–39], which is often not (yet) the case.

Patients with HF typically have multiple comorbidities [40] and crossover symptoms (e.g. breathlessness in angina, chronic obstructive pulmonary disease, pulmonary vascular disease, anaemia, uncontrolled arrhythmia) [41]. In addition, certain clinical signs (such as tachycardia) may be symptom-generating in themselves (breathlessness), may reflect an underlying pathophysiological process (such as infection or fluid overload), or may even be the underlying cause of HF, which itself may also result in similar symptomatology. Teasing out these subtleties by history and physical examination can be extremely challenging, even by specialists [42]. Therefore, better stratification of patients with chronic diseases is essential in order to define personalised profiles that are required to achieve the paradigm shift to PPPM.

#### Challenges in HF care

Choosing the wrong therapy may have potentially deleterious effects. Severe side effects and even death can occur, e.g. in patients wrongly deemed to be systemically congested due to inappropriate diuretic administration [43], or intensifying rate controlling medications in tachycardia related to congestion without addressing the underlying volume overload [44]. Equally, delaying *appropriate* therapy may reduce the efficacy of the intended therapy, e.g. early intervention with diuretic therapy to reduced volume overload is more likely to be effective whereas once systemic volume has been retained the oral route is less effective necessitating intravenous therapy [45, 46]. In complex fields with a broad library of research, case histories and potential therapies, it can be difficult for individuals to remain up to date with best evidence—especially in complex diseases where software such as the IBM Watson has shown some promise in guiding appropriate therapy [47].

Added to this is the concept of a patient acting as the gatekeeper of their own baseline health status and an active invigilator in detecting when their baseline has changed. This may be the ultimate trigger in deciding when to change therapy. A patient’s subjective awareness of what different disease processes feel, e.g. does chest pain feel like the angina previously experienced?, may be important in accurate diagnosis and treatment.

Computer-guided algorithms have the advantage of processing many data points quickly. However, they rely on accurate contemporaneous patient input and engagement. A computer may also be less accurate in certain elements of patient care, which may be difficult to quantify in an algorithmic fashion, e.g. patient frailty, difficulty to tolerate medications, wishes regarding ceiling of care and end of life care.

#### Therapy monitoring

Healthcare professionals and computers may be complementary to determine response to therapy. For example, clinical response to therapy may be earlier than objective measures of response such as biochemical [48] or weight [49] parameters in some but later in other patients. On the other hand, clinically obvious parameters may be obscured and alternative diagnostic tools may be more sensitive in detecting congestions [50]. Thus, remote diagnostics with appropriate analyses techniques may be superior to clinical findings; however, this might not be uniform in all HF patients.

Similarly, distinguishing side effects of medications from the underlying disease process and comorbidities may also be challenging for both machines and humans. Examples may include, does tiredness reflect low cardiac output or effect of treatment such as beta blockers? Does deterioration in renal function represent progression of the underlying HF, an expected response to angiotensin-blocking agents or overdosing diuretic therapy? It is possible that AI may be able to improve distinction, prediction, and prevention significantly, but this has not yet been properly tested.

These questions highlight the complexity of applying a structured diagnostic algorithm to the clinical course of a disease, as well as the inherent complexities in its personalised management. Given the broad phenotypic variation on both a personal and population level, it is likely that a combined approach of well-informed ‘baseline’ clinical algorithms along with personalisation to individual patients are needed for maximum efficacy. In particular, an AI-delivered therapeutic strategy needs to learn how the patient responds to treatment, how complications of both treatment and disease present, and be able to alter future responses based on accumulated knowledge—similar to how a physician becomes familiar with an individual patient over time.

### Participatory medicine: new roles for both patients and healthcare professionals

#### Patient-centred care

In recent years, the doctor-patient relationship has evolved beyond the paternalistic ‘doctor knows best’ model, toward one where the patients wish to be more actively engaged and expect a degree of input in their care [51–53]. This, in turn, represents an opportunity to not only involve the patient in decisions around their care but also to actively involve them in self-administering care.

Increased patient engagement in their own care has many potential benefits:

1. Improvement of patient adherence [54]

2. Improvement of patient satisfaction [55]
3. Reduction of direct patient costs (e.g. travel, parking) [56]
4. Reduction of indirect costs (e.g. productivity days missed for patients and carers) [57]
5. Improvement of patient outcomes and reduction of healthcare costs [58].

Patient-centred care represents a paradigm shift in how patients, doctors, nurses and other health professions think about the processes of treatment and healing. Defined by the US Institute of Medicine as the act of ‘providing care that is respectful of, and responsive to, individual patient preferences, needs and values, and ensuring that patient values guide all clinical decisions’, [59] patient-centred care prizes transparency, compassion and empowerment. The rise of patient-centred care makes way for a healthcare system designed to optimise the agency and comfort of the most important and vulnerable people in the equation: patients, their families and their communities [31].

**Fig. 2:**
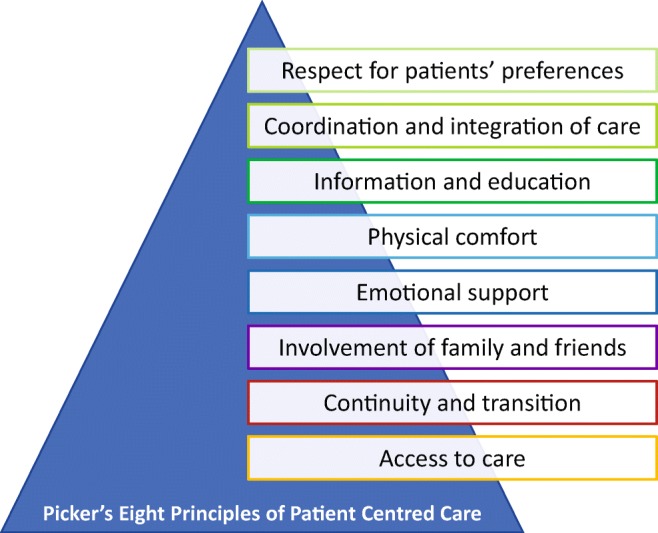
Picker’s principles of patient centred care, highlighting the most important elements of care to achieve high-quality healthcare with high patients’ satisfaction. These principles are important to integrate in the envisioned paradigm shift

Patient-centred care is a far-ranging new view of healthcare that resists simple summation. But there are a few consistent core ideas that guide this new style of care [60]. Researchers from Harvard Medical School, on behalf of the Picker Institute and The Commonwealth Fund, identify Picker's eight principles of patient-centred care (Fig. 2) [61]. However, as it turned out, the development from theory to practice is a lengthy and unruly process.

### Successful patient engagement

Current concepts imply that key to the success of patient engagement in self-care is educating patients about the clinical manifestations of their disease process, effects of medications, lifestyle modifications required and clinical particulars regarding the rationale for a prescribed treatment regimen [62]. However, for patients or their carers to truly take charge of day-to-day disease management, they must also be sufficiently supported and equipped with the essential tools, e.g. to deal with a change in clinical status—such as a structured approach to identification and management of deterioration, which may be ASYMPTOMATIC (detected by clinical measurement tools) or SYMPTOMATIC (which may be recognised by the patient or carer).

Detection of asymptomatic deterioration would effectively take the place following a physical examination performed in the clinic and may involve the use of simple measures such as weight and blood pressure, but may also expand in suitable patients to involve more advanced metrics such as implantable sensor data (e.g. CardioMEMS™ [50], OptiVol™ [63]). Effective monitoring may be improved by novel eHealth technology and sensors, which is a rapidly growing field.

Detection of symptomatic deterioration would take the place of ‘doctor-initiated’ history taking. So far, key to this process is educating and coaching the patient and carer in awareness of symptoms indicating deterioration of their condition, e.g. worsening breathlessness in HF [64]. It would also require educating the patient about symptoms, which may indicate an alternative pathology warranting alternative treatments, e.g. cough with sputum production in the setting of worsening breathlessness may indicate an infective process rather than deteriorating HF. However, telemonitoring can be more efficient than education alone in supporting patients and carers in detecting and interpreting symptoms [30]. In particular, the majority of patients with chronic diseases who are usually elderly may not be able to detect and interpret symptoms sufficiently without additional support.

Additional measures are required for a successful personalised self-guided care strategy. There must be (i) a relatively simple treatment algorithm in place to respond to changes in clinical status which the patient can follow, and (ii) backup from a clinical specialist nurse or physician if needed. Basic therapies, such as adjustment of diuretic doses [65] or adjustment of anti-hypertensive medications [66], may assist in preventing a hospital admission with acute deterioration. Modern eHealth technology using AI may be more efficient and may allow a much larger proportion of the HF population to apply personalised self-guided care strategies.

#### Supporting technology

New technologies enabling data generation by the patient will increase the data volume, which will improve timely information and education for patients and doctors in personalising therapy and predicting response in a personalised manner. This can also be enhanced by different approaches including the use of gameful design of support systems [67] to increase patients’ engagement in therapeutic processes and adherence and help identify and address their requirements and preferences in the context of self-management of chronic conditions, like HF. In turn, this will offer new ways of identifying patients early with very high likelihood for acute deterioration and death. One example might be that early indicators like changes in daily behaviours may prevent fatalities of individual patients by an early alarm system [68]. Remote patient management may detect early signs and symptoms of cardiac decompensation, thus enabling prompt initiation of specific treatment before full manifestation of HF decompensation. Ideally, this is initiated by the patients themselves instead of being professional driven, which is currently the case in telemonitoring systems. However, this means that patient participation, and their perspectives, need to be included in the development of new technical devices and healthcare approaches. This new approach in patients with a life-threatening chronic disease will be a milestone for approaches in integrated care, PPPM and managing patients with multimorbid diseases.

Therefore, the uniqueness of such novel strategy aiming to be a comprehensive patient-centred clinical support system for taking care of patients with HF, can also become a blueprint for managing patients with different other chronic diseases using eHealth solutions via intelligent algorithms. This will pave the way for overdue advancements in daily medical practice, namely, *improved efficiency in patient care coupled with high potential for cost reduction.*

### Support for changes in the care process using eHealth

There are numerous definitions for eHealth in the scientific literature. In short, eHealth is often defined as the use of information and communication technology in healthcare. In the context of this strategic paper, a more comprehensive definition is used; ‘eHealth is an emerging field in the intersection of medical informatics, public health and business, referring to health services and information delivered or enhanced through the Internet and related technologies. In a broader sense, the term characterises not only a technical development, but also a state-of-mind, a way of thinking, an attitude, and a commitment for networked, global thinking, to improve healthcare locally, regionally, and worldwide by using information and communication technology’. [69]

The development of new eHealth products is evolving fast, with the immense availability of healthcare related applications. These developments are not only supported by the industry who participate in their development, but also by patients, national and international societies of physicians, and governments [70, 71]. A vast majority of patients are willing to engage in these new practices with high patient satisfaction and improved health outcomes; these are the beginnings of a fundamental paradigm shift in healthcare [70]. A consultancy agency that explored the potential of medical technologies with regard to patient centred care needs, by order of the Ministry of Health, Welfare and Sports (VWS) of the Netherlands, concluded that the attitudes of patients and healthcare professionals for these new technologies are positive, but there is a reluctance in full adoption [70]. In addition to a lack of well-established evidence and reluctance of healthcare professionals to redesign care processes, insufficient funding by healthcare insurances or by Government [72], and the difficulty of linking existing information and communications technology (ICT) systems with the new technologies [70] are important barriers. The minister of VWS of the Netherlands has set up actions to encourage insurance companies, healthcare organisations, stakeholders and developer to cooperate and create a successful implementation and upscaling of the use of eHealth [71] indicating the support from the government for changes in the usage of eHealth in the care process. The expectation worldwide is that eHealth will be the way forward in achieving solutions for sustainable healthcare [73].

#### Patient-interface: how to facilitate eHealth for patient centred care

eHealth technologies are playing an increasingly important role in providing internet-based disease management, including self-management support, facilitating information exchange among professionals and with patients, and monitoring the performance of disease management programs.[74] In the approach of Eysenbach [69], eHealth is seen as more than just a sophisticated tool: it is about co-creating and evaluating an infrastructure for knowledge dissemination, communication, user engagement and the organisation of care with all involved stakeholders—patients, doctors, nurses and technology developers. This requires careful embedding of technology into the care process, with attention on added value in a dedicated context. Co-creating sustainable eHealth technologies thus requires a holistic development and evaluation approach that takes into account the triad between technology, users and context of implementation [75].

Patients newly diagnosed with a chronic disease are often overwhelmed with their situation. They feel insecure, sometimes anxious and often confused by the quantity and diverse provision of information. Affected patients have to learn to deal with their new living conditions, including the reaction of their relatives. Thanks to new technology it is possible to offer them remote support and online coaching with which they can complete efficient self-care. Practice has shown the importance of relevant knowledge for personal processing of a diagnosis, behavioural improvement and timely recognition of symptoms to prevent exacerbations. The mission of eCoaching is to reach optimal HRQoL. ‘Patients make better medical choices with coaching’, emphasises Jeff Belkora, founder of Patient Support Corps and Professor of Surgery and Health Policy at the University of California.[76]

Usual care for chronic diseases has been limited to episodic visits to various health services in which interim results and events are reviewed, and plans are made for adjusting care as needed, based on the information acquired during the consultation. In order to unburden both patients and healthcare professionals, modern care should be provided at the convenience of the engaged patient, taking advantage of modern technology. Against this background, the concept of eCoaching in care of chronic diseases may help to optimise patient independence and outcomes using eHealth. The combination of the various aspects of eHealth including remote patient monitoring, eCoaching, and decision support enables patients to be treated outside of conventional clinical settings, potentially improving HRQoL, engagement and compliance, and outcomes in chronic diseases.

#### Gamification to improve patient engagement

Incentives and rewards are one of the major means to attract users’ attention and ensure long-term engagement. Through proper and carefully thought through design, reward programs can be of high importance, as they provide (necessary) motivation for behaviour change and better long-term engagement. There are two elements of a reward that need careful planning; timing and nature of the reward. Ultimately, the goal is to maximise motivation and effort prior to receiving the reward, as well as happiness after receiving the reward [77]. Gamification employs a seamless combination of a number of mechanisms, tailored to specific situations on a case-by-case basis in which users find themselves, taking into account their personal values and expectations.

Success of gamification is driven by various factors, which include emergence of mobile devices, rise of big data and wearable computing [78]. Gamification in combination with AI found its use in various contexts including intelligent tutoring systems, health applications with decision support systems and a number of industrial applications that benefit from gamification mechanics and dynamics elements [79]. Thus, the inclusion of gameplay elements and systems into non-game contexts, such as healthcare, education, training, and work can enhance the user experience, and encourage behaviour modification and long-term participation. Various stakeholders including healthcare providers are investigating how to ‘gamify’ their processes and systems to increase engagement [80]. Some examples include the use of gamification to run a global awareness campaign to prevent cancer [81], and the DIDGET Blood Glucose Meter, that plugs into a Nintendo DS or DS™ Lite gaming system to reward kids for consistent testing [82].

Gamification can also be used to provide engagement to use AI based platforms. The growth of digital media has seen an explosive growth in the exchange of data, with its ability to collect, store and sort data, that is often an impossible task for humans. Crucial to the success of AI, are the algorithms that can learn and advance their intelligence. One way to inform these algorithms is to use gamification. Games are mainly complex systems, but tracking and storing data from them is easy and straightforward. Still, such an approach not only enables AI to learn and to improve, but also brings benefits to the user, who is motivated to engage with the application through the various gamification features.

Gamification can be used to track and measure progress. Using the various elements mentioned above, stakeholders can store and track the performance, progress and aptitude, and can make further decisions based on this data. In healthcare, this may result in using gamification and AI for diagnostic purposes. There are various benefits of using gamification to provide engagement in AI-based platforms, such as [83]:

1. Flexibility in addressing problems and challenges
2. Knowledge being more transparent
3. More data for AI to learn and improve from
4. Low-cost and low maintenance required for AI.

Eventually, this will result in better patient adherence to using of the eHealth application and simultaneously to gather additional diagnostic information to better monitor and guide patients. Uniquely, via data analytics, AI can learn how best to enhance patients’ self-care capability. Thus by combining AI and gamification, an eHealth application may be smart, intelligent and entertaining at the same time.

### Understanding the role of *artificial intelligence* in the paradigm change

When a machine mimics ‘cognitive’ functions that humans associate with the human mind, the term ‘artificial intelligence’ is applied. Examples are data analysis and prediction, image analysis, human speech recognition, self-driving cars and many others. Tools in AI are based on statistics and specific methods like logistic regression, neuronal networks (deep learning), random forest, ensemble tree, fuzzy partition, support vector machines, self-organising maps and more. Many AI algorithms are capable of improving from data, to enhance themselves, by learning new strategies that have worked well in the past. AI algorithms consider possible hypotheses and match it against the data [84–89].

Supervised learning means learning a function that maps an input to an output based on example input-output pairs. Therefore, a supervised learning algorithm analyses the training data and produces an inferred function, capable for mapping new examples [90]. Unsupervised learning stands for inferring a function that describes the structure of data that has not been classified or categorised. In this case, there is no straightforward way to evaluate the accuracy of the result which has been produced by the algorithm.

McKinsey predicts healthcare as one of the top 5 industries with more than 50 use cases using AI [91]. AI will change the healthcare industry, support doctors in improved provision of healthcare and empower patients in controlling and maintaining their health. AI is perfectly suited to support PPPM. There exist already many examples which provide a clearer view as to how AI will affect healthcare. The direction of impact is inclusive of making hidden information into data usable, gain of efficacy and efficiency, risk identification, prevention, triage of patients, telehealth, virtual nursing, robot surgery, therapy guidance, self-treatment, higher precision, prediction, personalisation and relief from routine tasks.

Complex chronic diseases like HF require personalised diagnostics and therapy, which may be supported by AI to improve prediction, therapy guidance and prevention of deterioration. Nowadays, diagnostic companies still focus on approaches using a limited number of markers, leaving interpretation to the healthcare professional. However, the dynamics of complex diseases are often only determined by the use of a broad range of markers using different diagnostic modalities. In contrast to the human brain, AI is able to deal with the exponential increase of possible combinations related to multi-marker approaches. Therefore, the paradigm change using AI requires the availability of a broad range of data, including patient history and characteristics, signs and symptoms of not only heart failure but also accompanying comorbidities, results of diagnostic tests, input from multiple markers (e.g. biomarkers) and sensors, medication and other aspects of treatment. In essence, the algorithms require the same information as healthcare providers use for decision-making.

AI introduction in healthcare is starting with hybrid models. Physicians are supported in diagnosis, treatment planning, identifying risk factors, but retain ultimate responsibility for the patient’s care. This will result in faster adoption by healthcare providers by mitigating perceived risk and delivering measurable improvements in patient outcomes and efficiency at scale. Long term, there will be a shift from decisions exclusively made by physicians and other healthcare providers to a hybrid decision model, where unambiguous decisions are made by AI and directly advised to the patients but leaving the responsibility for more complex or ambiguous decisions to the healthcare professionals.

### Expected “added value” of AI services in healthcare and potential harm

The face of modern healthcare is changing dramatically and AI has a significant impact on this in two main ways; *assisting clinicians in the delivery of care* and *the extraction of meaning from ‘Big Data’* [92]. Task delegation and sharing is becoming significantly more important with the global shortage of healthcare professionals and the increasing number of patients. AI may reduce healthcare expenditure and ultimately improve quality of care. A prospective analysis concluded that the use of AI applications could *save American healthcare by approximately $150 billion annually by 2026* [93].

Despite several examples of AI already successfully introduced in clinical practice, adoption is not yet broad, with some exceptions, and implications to clinical practice is limited. This will, however, rapidly change as the following examples illustrate. AI has been ‘visible’ through clinical decision support systems (CDSS), in care delivery across specialties. Castaneda et al. diagrammatically illustrated a typical clinical consultation and the effect a CDSS may have [94]. The amalgamation of information to support clinical decisions was publicly evident in IBM’s ‘Watson’ [95], which had the capability of replicating up to 90% of the decisions made by healthcare professionals. In terms of delivering personalised medicine, AI has a distinct advantage in its ability to pool massive data sets and extrapolate their relevance to an individual—as evidenced in a novel program designed to calculate a woman’s ‘Hereditary Breast and Ovarian Cancer’ risk result and offer recommendations and questions for her to raise with her healthcare provider [96].

Furthermore, the algorithm for the detection of cardiac rhythm changes (ST elevation) is commonly available with the majority of 12 lead ECG machines offering analysis and enabling the provisional diagnosis of myocardial infarction and cardiac arrhythmia [97]. Other recent work has demonstrated the ability to predict risk of cardiovascular disease based purely on a still image of a patient’s retina [98]. An AI-based test of multiple blood derived biomarkers detected coronary artery disease with an accuracy which so far was only achievable by advanced imaging technologies such as modern contrast CT angiography [99]. A mobile Atrial Fibrillation App incorporated clinical decision-support tools alongside educational material, patient involvement strategies and follow-up [100]. Patients reported the app was user friendly and significantly improved patients’ knowledge, drug adherence and anticoagulant satisfaction, increased quality of life, and reduced anxiety and depression. Indeed, AI has the capability for real-time continued monitoring (whether in the home setting or within healthcare settings) with built-in care pathways for escalation and interventions when needed, ultimately empowering patients in self-management to improve clinical outcomes [101, 102].

Further advancements in AI have led to the development of embodied conversational agents or avatars, which can further improve user engagement and effectiveness of an intervention, for example directing patients to the most appropriate care service. Since availability is 24/7, avatars can monitor patients and provide quick and timely answers. Thus, integrated AI technology provides health state analysis, decision support and treatment recommendations. For example, an animated conversational agent playing the role of a health counsellor, through a series of daily conversations over two months, had a positive impact on fruit and vegetable consumption [103].

AI may also improve the diagnostic process of many diseases. A recent Stanford University study tested an AI algorithm to detect skin cancer against dermatologists, and found it to perform at the level of the humans. Further, Baidu Research announced that the results of its deep learning algorithm can outperform humans with identifying breast cancer metastasis [104]. AI is providing several solutions for triage of patients that provide quick, scalable access for basic questions and medical issues. Additionally, unnecessary trips to the GP can be avoided, reducing the rising demand on primary healthcare providers and providing basic guidance that otherwise would not be available for populations in rural areas. In radiology, AI solutions can automate image analysis and diagnosis. These solutions drive efficiency and reduce human error. Improved tumour detection on magnetic resonance imaging (MRI) and computed tomography illustrates the progress. The US Food and Drug Administration (FDA) has given clearance for an AI platform which analyses and interprets Cardiac MRI images. For humans, image analysis is very time consuming. A Massachusetts Institute of Technology led research team developed a machine-learning algorithm that can analyse 3D scans up to 1000 times faster than before, making it possible to study changes almost in real time [105].

‘Big data’ involves the integration and interpretation of large volumes of healthcare information, such as biomedical and clinical data to generate robust scenarios applicable for everyday life. In the UK, information from electronic health records has been used to inform prediction of drug effects and interaction, identification of type II classifications and discovery of comorbidity clusters in autism spectrum [106]. Furthermore, there is increasing evidence on the benefit of computer-based decision support within pharmacology with the ability to reduce physician errors and quicken decision-making, thereby potentially saving lives. AI may also help to identify new potential therapies from vast databases which could be redesigned to target critical threats. This could improve the efficiency and success rates of drug development, accelerating the process to bring new drugs into market.

AI-assisted robotic surgery gets an increasing amount of attention. At present, Da Vinci is the most advanced surgical robot that allows doctors to perform complex procedures with greater control than conventional approaches. Heart surgeons are assisted by heart lander, a miniature robot that enters a small incision on the chest to perform mapping and therapy over the surface of the heart. Machine learning has been instrumental in orthopaedics, in terms of understanding biomechanics, orthopaedic implant design [107], prediction of progression of osteoarthritis [108] and robotic surgery [109].

A number of future avenues for the use of AI in the ‘big data’ domain includes the use of unsupervised learning techniques to more precisely phenotype complex disease [110] and facilitate earlier prediction of an epidemic. It is anticipated that the World Health Organisation (WHO) will be able to monitor big data in real time from a wide range of international sources, thus mitigating the progression of an epidemic [111]. AI can already address healthcare challenges within resource-poor settings, for example Onu described signal processing and machine learning in Nigeria, whereby mobile phone data has been used to predict birth asphyxia [112].

The large potential of AI for improving healthcare is indisputable. The question is how to integrate it safely and successfully into our everyday life and healthcare systems. According to the authoritative online publisher The Medical Futurist in the e-book ‘A guide to Artificial Intelligence in Healthcare’ a range of hurdles must be tackled before AI redesigns healthcare.

#### Potential harm and hurdles of AI introduction in healthcare

It is the promise of AI has to be balanced by possible risks and harms. One of the greatest concerns of introducing AI into healthcare is the potential for error and fraud. Despite an increasing emphasis on healthcare quality and safety, medical errors are not uncommon and pose a serious public health problem. Indeed, hospital medical errors are the third leading cause of death in the USA [113]. AI is being used or trialled for a range of healthcare and research purposes and though it has the potential to address important health challenges, its use also raises ethical issues such as the potential to make erroneous decisions, compromise or fail to safeguard patient health data, and be used for malicious purposes [114]. For example, in using AI, medicine may be susceptible to adversarial attacks both in terms of monetary incentives and technical vulnerability, subsequently caution in deploying AI in clinical settings has been urged [115].

Embracing advances enabled by AI is likely to incur cost savings and efficiency. Whilst it may be argued that AI might be expected to make healthcare safer and more efficient, we need to ensure that our datasets are robust, have continuation of reliable data supply, and have sufficient breadth and depth for accurate diagnosis [116]. However, healthcare is still under-digitised in many countries. Many primary care physicians still keep their medical records on paper (e.g. Germany 34%, Switzerland 41%) [117]. In many countries, the digitisation of the health system corresponds to the level that financial services and industry had more than 20 years ago. While in other industries digital tools are widely used and decision support tools are seen as essential aid, some doctors do not yet see the added value, with potentially tremendous negative effects on productivity and accuracy.

Along with a wealth of information comes responsibility inclusive of issues of privacy, ethics, data security and accountability. Although AI can virtually remove man-made error from processes, it can still exist in the programming: being largely algorithm based, the technology is not exempt from bias or prejudice [118]. Hence, AI is only as intelligent and discerning as those responsible for its initial programming, noting that later improvement by self-learning algorithms may be possible. Currently, there is a dearth of regulations and lack of standards to assess the safety and efficacy of AI systems. However, the FDA has made some inroads in an attempt to provide guidance for assessing AI systems [119]. Further to this, by succeeding in creating ethical standards, developing measures of success and effectiveness and by making it available to the mainstream, we can reduce many of the privacy concerns and misapprehensions surrounding AI [120].

Perhaps one of the biggest trepidations about AI is that it will become so sophisticated it will take over our lives. Is there the potential for AI to take control away from humans, de-humanise actions, reducing compassion and empathy? On the other hand, the complex and unique circumstances surrounding HF often require clinicians taking judgement calls into account when making decisions; as such the question then becomes, *Can AI be programmed to successfully manage complex long-term conditions*? The most probable answer to this question would be: yes, in part.

Clinicians need to be prepared for a future where their intellect and clinical discernment can be replaced—at least in part—with systems that are much more robust. This also means that the tasks of the healthcare professionals will change. We need to acknowledge the benefits and identify how best to cope with any perceived or real drawbacks of AI in the management of chronic conditions such as HF. For successful integration in the care process to enable PPPM, close ‘collaboration’ of technology using AI, complex algorithms, avatars and clinicians will, therefore, be key.

### Towards a cost-effective and sustainable economic model for integrated HF care

As argued above, in an ideal situation of healthcare maximum effort should be invested into (1) facilitation of therapeutic decision making for and by the patients along with their local carers in a home setting, while taking social factors into account; (2) early detection of critical cases and/or new disease(s) which require interventions by patients or specialist time and hospital capacity, to ensure optimal use of these scarce resources; and (3) prevention at home, promoting healthy lifestyle choices especially for those who are predisposed to chronic non-communicable disease conditions.

To achieve this, a paradigm shift in healthcare is needed, which is now possible from the advances and new developments in AI evidence-based medicine. Innovation in medical diagnosis and treatment is bringing new opportunities to change the landscape of evidence-based medical treatment. In practice, AI is not one new technology, but a variety of technologies, algorithms and software tools. While the clinical advantages seem to be obvious, there are a number of issues that need to be addressed and overcome for future sustainable improvement in patient health outcomes.

#### Expectations vs commercialization and sustainability

Having a new technology or device developed and tested in a controlled environment (laboratory or hospital with carefully selected patient groups) does not mean that it is ready for use in a wider ‘real world’ context. For broad exploitation in the healthcare market, several issues need to be addressed:

- Personalised technology or device is not fully validated or not applicable to or not tested in the relevant context—e.g. decision making not applicable to the specific contexts; poor quality (low signal to noise ratio), poor prediction, lack of robustness (drop outs, maintenance issues).
- Accessibility cannot be guaranteed—e.g. distribution problems, price too high, device not certified, no reimbursement, lack of knowledge by care professionals of its use.
- Despite advances in new AI-supported systems, proper clinical validation is still essential—e.g. one aim of validation is to prove the benefit but also to identify and address flaws and biases of the system.
- Implementation in clinical care is mostly lacking and not properly tested—e.g. several additional challenges need to be addressed to enable commercialisation.

Some of these aspects are specifically related to AI based systems, others are of a more general market-based nature. Several additional challenges need to be addressed to enable commercialisation:

- Regulatory authorisation—procedures may evolve much more slowly than the technology developments in this field and acceptability of a new personalised approach may be limited.
- Ethics—what do we allow the AI system to handle, decisions will have to be agreed and incorporated into (ethical) guidelines and contracts.
- Responsibilities—decision-making procedures need to be adapted, professional liabilities should be well described, perhaps even insurance coverage expanded, hence legal input is essential.
- Social challenges—resistance by medical staff to change their work practices, overcoming perceptions that AI may replace/change jobs, resistance by patients and their carers (accepting as substitute for care professionals, which type of advice is acceptable, e.g. lifestyle changes, therapy decisions), institutional resistance to change (need to change processes) and practical difficulties (specific training required, investments to be made, risks taken).
- Safeguarding—the position of patients as vulnerable adults and safeguarding the involvement of carers in the AI-supported systems.
- Skills—specifically, trained staff who can manage, maintain and/or work together with AI-supported systems. Also, the patients and their carers will need education on how to optimally benefit
- Financial—the financial structures, accounting conventions, financing availability and health reimbursement models involved vary by region or country.
- Legal—governance and data management to maintain patient confidentiality and manage data exchange in appropriate ways. The use of Common and Civil law in different countries across Europe may require the use of a range of legal solutions that may differ country by country.

#### Valuing social-economic impact

The discussion about who bears which costs is one of the most basic elements in developing a sustainable Business Model in healthcare commissioning and reimbursement. Investing in preventive measures and improving HRQoL in their home setting makes a lot of sense and can have a significant economic impact. However, quantification of socio-economic impact may be a challenge (for lack of generally accepted indicators and standardised methodologies); *Which participant in the health commissioning framework will realise the very real financial benefits that will accrue over time and therefore be able to accept the cost of provision?*

A more fundamental underlying issue is to focus on health outcome and long-term impact. This is especially true when seeking to improve the access to long-term healthcare treatment and quality of life for patients who require chronic care. This goes beyond the more common challenges of commercialisation in the healthcare sector and requires acceptability by all involved stakeholders. Sustainability in this context requires insight into the financial/economic context. It is important to develop a specific Business Case for these integrated and personalised systems considering the different routes to commercialisation, which depends on who is the consumer, who actually is paying for it:

- Patient—can pay for a sensor or smart phone App, but paying the full price to access an integrated therapy support system may be beyond a patient’s reach unless the AI-system lowers the access fee to a reasonable level (volume pricing).
- Care provider—a group of practitioners provide specific tools or aid devices for home care or a clinical centre buys diagnostic or imaging equipment (often the quality is the first indicator).
- Insurance company—a device, therapy or care service is reimbursed (often cost reduction is the first indicator).
- Community—a city, region or other commissioning area decides to provide certain forms of healthcare (often negotiated as package) to all patients in their region (volume pricing).
- Government or intermediary bodies—after formal assessment (health technology assessment (HTA)) access and prices for (a package of) certain products, therapies or provision of services with are recommended, facilitated or established as sole choice (formal tendering process). Obviously, combinations of these are common.

Two other elements are integral to sustainability, i.e. scalability, achieving critical mass so that prices can go down, and transferability from one environment to another environment. Unfortunately, across Europe there is a huge variety of structural conditions, and the legal, regulatory, governance and reimbursement requirements, opportunities and constraints on health budgets are quite different. The regulatory frame is hindering healthcare development towards higher efficacy. Guidelines are one example that does not even consider the use of AI. Guidelines are history-oriented and do often not meet the requirements of modern medicine. A report by the US Institute of Medicine suggested that it takes on average 17 years before new knowledge generated in randomised trials is incorporated into practice and even then, acceptance varies considerably among centres [121]. Novel AI-based technologies may help to facilitate implementation of novel therapy but may face the same lack of acceptance even if validated in clinical studies.

In developing a sustainable, scalable and transferable AI-based solution all these factors must be comprehensively considered in early development, recognising that many of the building blocks are at different stages of technical, operational or social development. Failing to do so may result in restrictions on the Business Case, significantly impact the required balance of (co-) financing and reduce the potential level of technological innovations that are implemented in the market. This will in turn restrict the application of AI across the healthcare market perspectives and its consequential long-term growth potential.

The bottleneck is, therefore, not necessarily the technology (although new developments may open up new approaches, as discussed above). It is essential to involve all stakeholders at an early stage for proper validation and implementation to enable financially sustainable models with lasting impact on our healthcare system and on the patient’s life. In fact, it is probably true that a mediocre technology pursued within a great business model may be more valuable than a great technology exploited via a mediocre business model. Unless a suitable model can be found, these technologies will yield less value to the firm than they otherwise might [122].

### Legal, ethical and societal issues

As the acceptance of AI grows, so do the ethical and societal questions concerning its implementation into the health system. The future impact of the new technology provides various challenges. Possible ethical, legal and societal issues could arise, e.g. in regard to a possible discrimination on the part of an AI system and the question of accountability, in the case of mistakes and the patient-physician relationship.

An important ethical issue is the potential for AI to unfairly discriminate between patients, coming from the training data that contains human biases [123]. Thus, a recent study found that some facial recognition programs incorrectly classify the gender of less than one percent of light-skinned men, but more than of one third in dark-skinned women [124]. The most widely used cardiovascular risk score developed using data from mostly white patients may be less precise in minorities [125]. Further, most evidence-based treatment recommendations in HF result from studies in white men [126]. An uncontrolled AI algorithm could make therapeutic decisions based on preexisting biases, especially when used for complex conditions with a high degree of uncertainty. In HF, this may be particularly true in patients in the palliative phase of the disease course or in patients with HF and preserved LVEF, where solid evidence is lacking. The clinical consequences of such potential misinterpretation are not known. Still, clinical decision-making faces the same biased evidence, where we accept extrapolation without support by evidence [126]. Thus, it will be important to include testing routines for detection of potential bias in programming of AI and in critically reviewing the results of it to achieve true PPPM that is superior to current care. In addition, circumstances need to be considered where AI may not provide the required results and human intervention is required, such as in the palliative phase of care.

Associated with this arises the question of accountability. When an AI system fails at a certain assigned task, who should be responsible? The programmer, the data owner, or the end user [127]? The question of who is responsible if AI makes a mistake is still unanswered [128]. Self-driving cars provide an example. Those vehicles could be involved in accidents, just like human drivers today. The difference is that we have a clear understanding of fault and blame for human drivers, but this does not yet exist for AI. A car could be programmed to act in the safest way for the passenger, or it could be programmed to protect the people in the other vehicles. Whether or not the manufacturer or the owner makes that decision, the responsibility for the fate of people involved in a car crash is not yet resolved. The same principle can be applied to AI systems in healthcare. The scope and content of these restrictions, e.g. whether and how AI can be intelligible and will apply, remain uncertain and contested [129]. Questions of accountability and liability are easier to answer when the reasons that lead to a certain decision or action are comprehensible. Therefore, transparency is another aspect that needs to be considered when discussing AI [129]. AI algorithms may be seen as a kind of ‘black box’ [130]. Thus, AI is difficult to understand or interpret and it may be impossible to determine how AI has reached its decision. This could lead to bad adherence, e.g. if patients do not know why the AI system suggests a particular diagnosis, treatment, recommendation or outcome prediction [129]. Also, medical research has to be transparent, requiring ways to uncover and show the ‘inside’ of an AI algorithm. Considering the expected complexity of such algorithms, this will be an almost impossible endeavour.

Besides that, there are also important issues regarding data sharing and protection of AI system, which need to be addressed, taking the strict ‘General Data Protection Regulation’ of the European Union into account. What should be the code of conduct? What information is really necessary to ensure the best treatment for the patients, without exposing their sensitive data regarding their health? What could be the risks of automatic profiling of patients [129, 131]?

The impact of AI tools on the patient-physician relationship regarding the decision-making process may be an additional concern [132]. Shared decision making is considered an important prerequisite of this relationship. One difficulty is seen in the increasing availability of information on the internet, some of them with questionable content and limited scientific reliance [133, 134], and the increased use of this option by the patients [135]. The variation in quality of information or misunderstandings may generate conflicts between the patient and the physician and may require a significant amount of time to resolve [133, 134]. However, the implementation of AI systems developed by patients and physicians together may help to overcome this problem and may help patients to make well-informed decisions about complex medical issues. Another way could be quality regulation or an evaluation and certification program for AI systems and health technology in general [136]. Thus, AI-driven decision support tools can significantly change the way treatment decisions are made. Importantly, to assist in achieving sustainable healthcare systems the relationship of patients and physicians needs to be redefined and the future role of physicians clarified [132, 137].

Several studies have shown that good doctor-patient relationship positively effects health outcome [138–140]. Therefore, even if AI is successfully introduced in clinical practice human interaction may still be required in healthcare, particularly in areas with social interaction and a demand for a holistic perspective. However, the exact new roles of healthcare providers and their effect on patient outcome need to be tested. Diagnostics and therapy recommendation should also consider soft factors like patient fears and worries, the social environment, lifestyle and other conditions. It remains to be determined to what extent AI can address these ‘soft’ factors.

In view of the imminent collapse of the current healthcare system, relieving physicians of administrative and routine tasks could be the key [141]. These tasks could be performed by AI systems, leaving more room and time for physicians to spend time with their patients and being empathetic. In addition, some specific medical professions, such as radiologists, could in future be replaced (in part) by algorithms able to interpret images even better than human doctors [142]. However, it is very unlikely that AI will completely replace allied professionals in the health sector, yet doctors who use AI will likely replace those who do not [142]. Therefore, knowledge and a basic understanding of the key principles of AI systems will need to be a crucial part for the future generations of all healthcare professionals.

### Who are the beneficiaries of the novel approach?

The proposed PPPM concepts, based on multi-professional expertise, foresee a facilitated knowledge transfer between innovative sciences and advanced medical services, and incorporate strategies considering interests of:

- Individuals in suboptimal health conditions predisposed to chronic pathologies such as HF—by personalised innovative screening programmes and preventive measures tailored to the individualised profiles
- Patient cohorts—by precise patient stratification/prediction according to the disease subtype, risk factors, collateral pathologies etc.
- Individual patients and their relatives—by measures adapted to personalised needs, including treatment algorithm tailored to the person, self-monitoring and active involvement of the closest environment in the treatment process
- Healthcarers—by innovative educational programmes, digitalisation of routine procedures and decision-making process
- Diagnostic and pharmaceutical industry—by creation of extended market opportunities
- Biomedical sciences—by motivating innovative research in the context of PPPM
- Healthcare systems—by improved operation processes and positive economy
- Society as a whole—by advanced ethical and socio-economical solutions.

The complex measure of this PPPM approach may be considered as the medicine of the future [33]. Contextually, the management of chronic diseases benefits particularly from application of AI technologies. This is due to huge potential in machine learning, data processing, computation analysis, monitoring and treatment of complex and collateral pathologies such as HF, which represents a highly heterogeneous patient cohort and may strongly benefit from improved subtyping in order to better characterise its pathophysiology and to develop novel targeted therapies [143]. In this way, AI can help clinicians deliver more accurate care and protects patients against potential harm by treatment mistakes linked to the disease complexity [144]. AI is becoming a mandatory technology in clinical practice. Machine learning and big data analytics have been proposed specifically for cardiology for predicting individual risks and applying genomic information for precision medical approach. Currently, run projects employ machine-learning techniques to address the problem of classification of HF subtypes and unbiased clustering analysis using dense pheno-mapping to identify phenotypically distinct HF categories [145].

### The HF case report of the future

Although not experienced with computer technology, Mr. Johnson is using a physician avatar on a tablet computer. Access is available and data protection guaranteed by iris detection, which is so easy for him that he does not have to worry how to use it. Once weekly, he performs an outpatient visit by himself with the help of the physician avatar. The interval of the visits could also be longer depending on how stable his condition is, but he feels safer with this interval. The avatar advises him how to take medication, sends new prescriptions to the local pharmacy if required and tells him when to have his blood tested. To do this, he has to go to the local healthcare point in the village. However, he was told that in the near future, the avatar will be able to this at home. He already uses tools to measure his health—they call them sensors—and obviously, blood testing is going to be an addition to these tools. If he feels bad and has more symptoms, he can let the avatar do a check. Recently, he had more shortness of breath. The avatar advised him to take more diuretics and the symptoms disappeared rather quickly. After this episode, the avatar adjusted his treatment and now he feels very well. Before, there was an episode with very fast heart rate. The avatar told him that he needs to seek advice from his cardiologist because he had tachycardic atrial fibrillation. The cardiologist sent the ambulance to his home for a cardioversion. This went well and he felt much better.

The avatar gives him a lot of confidence and safety. He now has access to advice whenever needed at home. He has less contact with the healthcare providers but if he needs them, waiting time is much less. He gets advice regarding many other aspects of his daily life and has a much healthier lifestyle. In fact, exercise has become fun with the use of the avatar. He can hardly understand anymore, why he was so reluctant to use the avatar in the beginning.

## Conclusions and outlook

Healthcare cannot be maintained in its present form. On the one hand, rapid development of modern technology, particularly the broad implementation of AI as discussed above, will inevitably change healthcare. An ageing population, the accompanied increase in complexity of care as a result of the increase in comorbidities [26], and the expected decrease in healthcare providers, especially in rural areas [29], will result in large deficit of resources. Costs will escalate significantly and shortcomings in care of chronic diseases will become even more prominent than they currently exist [146]. Eventually, high-quality care will no longer be available for the entire population. To prevent these alarming trends and to ensure good quality care for all, the approach to care must change. The “one-size fits all” approach currently applied to patients with cardiovascular diseases must be replaced by a PPPM approach [147]. AI has the ability to help support such a paradigm change, but additional aspects need to be considered for successful implementation of this new approach to cardiovascular care. These may include the collection of real-world cohort data, sufficient description of individual patient characteristics and interventions applied, redefining outcome measures and new prediction models that are prospectively tested.

In order to achieve the necessary change in the management of chronic diseases, novel concepts are required, integrating the idea of self-care. The basis of such concepts must be the holistic view towards managing patients with chronic diseases, including the whole value chain. In contrast to the present situation in healthcare, patients need to be central in the chain, taking considerable responsibility within the care process. It requires important further steps that are getting more attention presently but are not yet sufficiently developed. These include among others a digitalisation in healthcare, multi-level diagnostics and therapy monitoring, disease modelling and an integrated care approach.

**Fig. 3:**
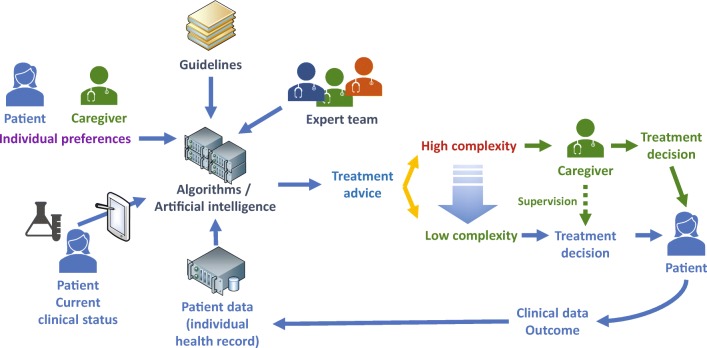
Novel hybrid concept for the paradigm change in chronic heart failure treatment including artificial intelligence (AI) supported self-care and targeted involvement of medical caregivers, requiring adequate multi-level diagnostics at patient side and comprehensive individual datasets. Depending on complexity, treatment decisions are being made by AI directly to the patients or by healthcare professionals (caregivers)

One solution, with high potential for reducing healthcare costs whilst maintaining quality of healthcare, is the transformation from solely professional provided care to AI enabled personalised patient self-care with shared responsibilities (Fig. 3). PASSION-HF is a project supported by INTERREG-NWE with exactly this aim. It will be achieved by collecting the needs of all stakeholders, particularly the patients, translating multiple guidelines into algorithms, linking these algorithms with AI and defining individual outcome models for patients with HF. The PASSION-HF consortium encompasses the required multidisciplinary expertise, including the medical field, patient knowledge, AI, telemedicine, serious gaming, data exchange and storage as well as business development and communication. The collaboration will not only result in new products to deliver care but also an increased acceptance of a new philosophy of chronic care. It will accomplish this by linking patient needs and technological innovation, in knowledge transfer to all stakeholders within the care process including patients and their relatives, and in new business models in healthcare. PASSION-HF will focus on HF as one of the most important chronic diseases, as well as addressing other important comorbidities. This will allow further expanding the concept to other chronic diseases after completion of the project.

## Abbreviations

AI: artificial intelligence
CDSS: clinical decision support systems
FDA: Food and Drug Administration
HF: heart failure
HRQoL: health-related quality of life
MRI: magnetic resonance imaging
PASSION-HF: Patient Self-care uSing eHealth In chrONic Heart Failure
PPPM: predictive, preventive and personalised medicine
VWS: Health, Welfare and Sport
WHO: World Health Organisation
